# Assessment of the Performance of a Portable, Low-Cost and Open-Source Device for Luminance Mapping through a DIY Approach for Massive Application from a Human-Centred Perspective

**DOI:** 10.3390/s22207706

**Published:** 2022-10-11

**Authors:** Francesco Salamone, Sergio Sibilio, Massimiliano Masullo

**Affiliations:** 1 Construction Technologies Institute, National Research Council of Italy (ITC-CNR), Via Lombardia, 49, 20098 San Giuliano Milanese, MI, Italy; 2 Dipartimento di Architettura e Disegno Industriale, Università degli Studi Della Campania “Luigi Vanvitelli”, Via San Lorenzo, 81031 Aversa, CE, Italy

**Keywords:** environmental monitoring, wearable devices, wearables, visual comfort, luminance mapping, glare, high dynamic range, HDR, low-cost sensor, do-it-yourself, open-source hardware

## Abstract

Ubiquitous computing has enabled the proliferation of low-cost solutions for capturing information about the user’s environment or biometric parameters. In this sense, the do-it-yourself (DIY) approach to build new low-cost systems or verify the correspondence of low-cost systems compared to professional devices allows the spread of application possibilities. Following this trend, the authors aim to present a complete DIY and replicable procedure to evaluate the performance of a low-cost video luminance meter consisting of a Raspberry Pi and a camera module. The method initially consists of designing and developing a LED panel and a light cube that serves as reference illuminance sources. The luminance distribution along the two reference light sources is determined using a Konica Minolta luminance meter. With this approach, it is possible to identify an area for each light source with an almost equal luminance value. By applying a frame that covers part of the panel and shows only the area with nearly homogeneous luminance values and applying the two systems in a dark space in front of the low-cost video luminance meter mounted on a professional reference camera photometer LMK mobile air, it is possible to check the discrepancy in luminance values between the low-cost and professional systems when pointing different homogeneous light sources. In doing so, we primarily consider the peripheral shading effect, better known as the vignetting effect. We then differentiate the correction factor S of the Radiance Pcomb function to better match the luminance values of the low-cost system to the professional device. We also introduce an algorithm to differentiate the S factor depending on the light source. In general, the DIY calibration process described in the paper is time-consuming. However, the subsequent applications in various real-life scenarios allow us to verify the satisfactory performance of the low-cost system in terms of luminance mapping and glare evaluation compared to a professional device.

## 1. Introduction

Glare is essentially produced by daylight or electrical sources and is essentially characterised by an uneven luminance distribution in the field of view (FoV) [1]. Glare can impair people’s visual performance or cause discomfort [2]. There are various indices for quantifying the glare in different situations—from the unified glare rating (UGR) used for artificial lighting to the daylighting glare probability (DGP) for light entering through windows to the contrast ratio (CR) defined by considering the contrast between certain luminance values and those of the surroundings [3]. In defining glare issues, the luminance of the glare source is, of course, the most important factor, but there are several other factors involved in the perception of discomfort, mainly based on the subjective adaptation level, which depends on the ability of the subject’s pupils to adapt to the light intensity [4].

Glare assessment could be based on the analysis of light distributions by luminance mapping, which allows rapid data collection in a large FoV. Low dynamic range (LDR) images are limited in the contrast ratio of the camera, i.e., the range of the light and dark parts of the image that it can reproduce. To overcome this technical limitation, it is possible to consider high dynamic range (HDR) images, which are created by taking and then combining several different exposures of the same scene. The main advantage of HDR is that it presents a similar range of luminance as that perceived by the human visual system. Although it is possible to create HDR using an absolute calibration method, there is also the option of using a stepwise method, which is described in detail in Ref [5].

Based on these premises, this paper aims to describe a do-it-yourself (DIY) approach to calibrating a low-cost wide camera connected to a Raspberry Pi microprocessor. In more detail, the study, which follows the dictates of the recent CIE 244-2021 technical report [6], intends to answer the following questions:

1. What is the response of a low-cost camera compared with a professional camera photometer in different controlled environments with different light sources?
2. Is there a considerable difference between the luminance values of the low-cost camera and the professional one, and is it possible to consider an eventually differentiated correction factor for the different lighting systems?
3. Eventually, is it possible to consider an even simpler algorithm that automatically adjusts the luminance distribution of the low-cost system considering the different lighting systems to adapt to that of the professional camera?

The method described in Ref [5] is time-consuming and cannot be performed automatically in a few seconds on a portable device. We would like to find out whether it is possible to limit the time for capturing the images to less than 3 s and how large the error is in the definition of luminance mapping, considering this important constraint and considering different light sources. For this purpose, we considered two cameras: a professional DSLR camera from Canon equipped with a Sigma fisheye and a Raspi cam controlled by a Raspberry Pi. These two devices were positioned in front of different lighting panels used as a reference luminance source (see the Materials and Methods section) to collect different data and check the discrepancy between the two camera devices used for luminance mapping. The main results of the study are then applied to different everyday scenarios to confirm our findings. The idea is to verify if it may be possible to attach the device to a helmet and capture information about the luminance level during the day from a human-centred perspective.

## 2. Materials and Methods

Two lighting panels were built, and different light sources (i.e., different light spectra) were considered on a small area with uniform luminance, as described in more detail in Section 2.1 below. Two luminance measurement systems were considered: one based on a low-cost approach and another on a professional reference instrument. For more details on the video luminance meters, see Section 2.2.

### 2.1. Lighting Panels Used as a Reference Luminance Source

**Figure 1:**
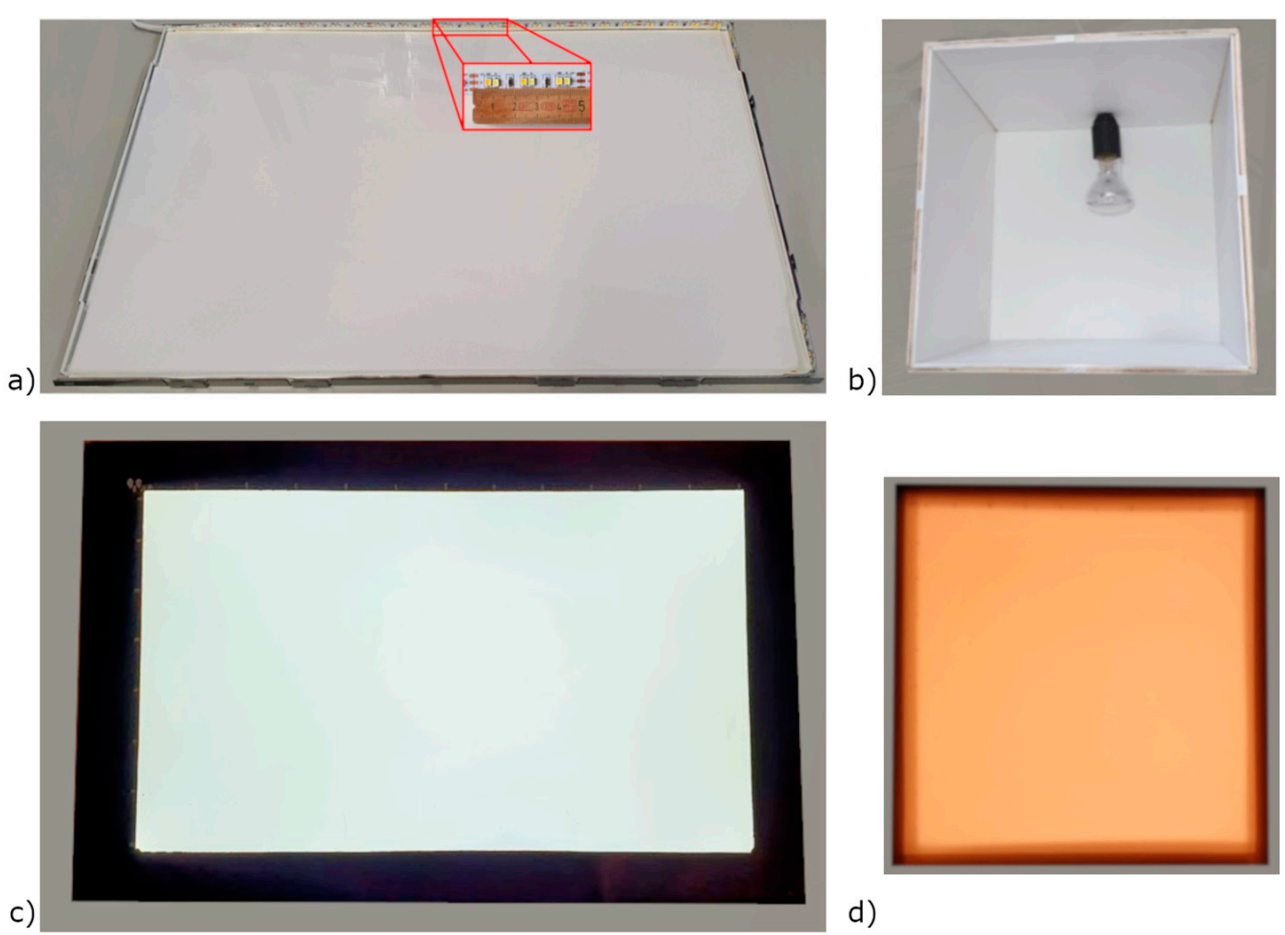
Luminance device based on a DIY approach: (**a**) LED panel as built; (**b**) wood cube with halogen E27 bulb lamp as built; (**c**) LED panel finished; (**d**) wood cube with warm white halogen lamp finished.

Two different lighting systems were developed for the luminance analysis, following the principle of the DIY approach. They consist of a LED panel and a cube with a standard E27 attack (Figure 1).

The LED panel is composed of different layers, from the bottom:

- An aluminium frame where the led strip was located on the long sides of the aluminium frame;
- An ethylene vinyl acetate EVA layer;
- A reflective paper;
- A light guide panel;
- A diffuser paper.

The strips consist of SMD2835 LEDs, both cool and warm white, spaced 1.6 cm (Figure 1a). A black frame is attached to the panel on which a Cartesian plane was drawn to define a mesh of points with a resolution of 3 × 3 cm (Figure 1c).

The cube, with external dimensions of 32 × 32 cm, is realised using laminated pieces of wood, with an inner cover made of white alveolar polypropylene and an E27 light bulb attack positioned at 6 cm from the bottom (Figure 1b), allowing the consideration of different lighting sources (i.e., halogen, fluorescent, incandescent). A foil of alveolar polypropylene was placed horizontally at 15 cm from the floor to reduce the luminance discrepancy on the test surface. The upper surface consists of a white synthetic glass panel. The same Cartesian plane with a grid of 3 × 3 cm points was drawn over this test surface (Figure 1d).

**Figure 2:**
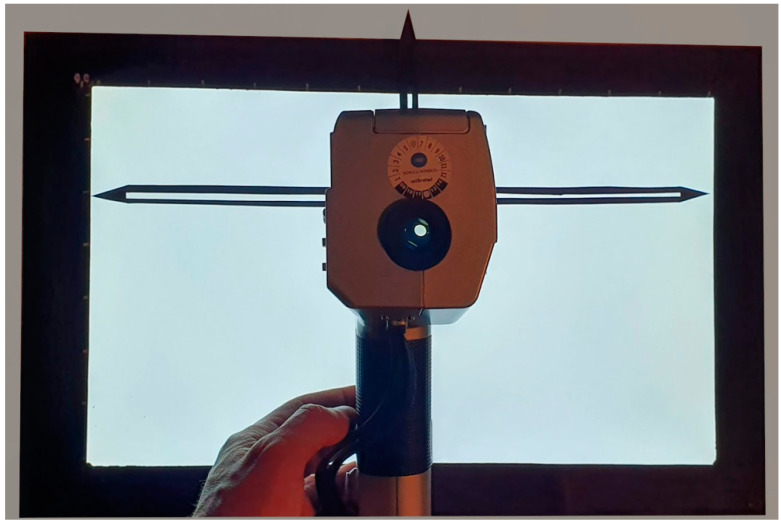
Example of characterisation of the LED panel (the same approach was used to characterise the cube).

A Konica Minolta LS-110 luminance meter is then used to evaluate the two panels’ luminance distribution, considering a template that follows the reference points across the x and y axes of the Cartesian orthogonal system (Figure 2).

The luminance values of the LED panel are defined in different configurations to allow CCT and intensity changes. On the other hand, only one configuration is considered for the halogen, fluorescent and incandescent lamp in the cube.

This approach made it possible to identify an area of the two plates with little differences in luminance distributions (see the details in Section 3.2 and Appendix A). In this way, it was possible to install masks on the 6 × 6 cm panels that limited the effective size of the lighting source, which was characterised by almost constant luminance and was useful for the subsequent analysis.

### 2.2. Equipment Used and Flowchart Used to Acquire the High Dynamic Range Images

The wide-angle camera with a focal distance of 1.67 mm, an optical FoV D of 160° (FoV H 122°, FoV V 89.5°) based on the OV5647 sensor, namely the V1 camera series, is considered in this research study. It has a native resolution of 5 MP and dimensions of 22.5 mm × 24 mm × 9 mm, making it perfect for mobile or other applications. The camera is connected to a Raspberry Pi 3 A+ equipped with a 64-bit quad-core processor running at 1.4 GHz, dual-band 2.4 GHz and 5 GHz wireless LAN, and Bluetooth 4.2/BLE [7]. The data collected by this device are compared with those of the camera photometer based on the Canon EOS70D digital single-lens reflex (DSLR) camera equipped with a CMOS Canon APS-C sensor and a Sigma Fisheye 4.5 mm F2.8 EX DC HSM [8]. Table 1 shows the most important lighting characteristics.

**Figure 3:**
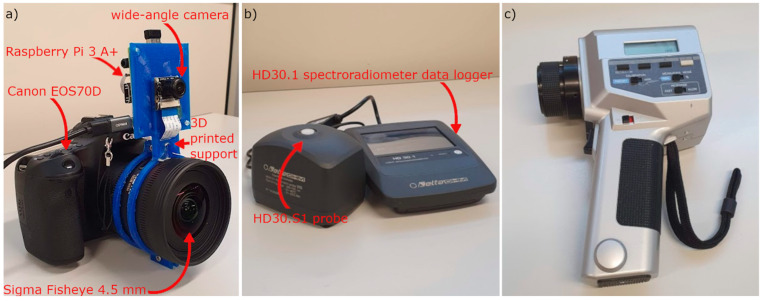
Equipment used: (**a**) reference camera photometer and low-cost Raspberry Pi with a wide-angle camera mounted on the 3D printed support; (**b**) spectroradiometer and light probe; (**c**) Konica Minolta luminance reference meter.

A 3D printed adapter was designed to install the Raspberry with the wide-angle camera on the fisheye lens of the DSLR camera (Figure 3a). The setup also uses an HD30.1 spectroradiometer data logger equipped with the HD30.S1 probe (Figure 3b) for spectral analysis of light in the visible range (380 nm–780 nm). It enables the calculation of the following photocolorimetric quantities: luminance (E) in [lx], correlated colour temperature (CCT) in [K], trichromatic coordinates [x,y] (CIE 1931) or [u’,v’] (CIE1978), colour rendering index (CRI_Ra) [9].

Both cameras took three different pictures of the same subject with different exposure times and combined them to create an HDR. The procedure for setting the shutter speed of the camera photometer corresponds to the A2 procedure described in Ref [10] and is based on the use of the hand-held Konica Minolta luminance meter (Figure 3c), which makes it possible to determine the correct time of high dynamic range (THDR). The procedure allows the measurement of the highest luminance value. The three “CR2” files collected with the camera photometer are then processed with LMK LabSoft to create the HDR file and generate a false-colour image of the luminance.

**Figure 4:**
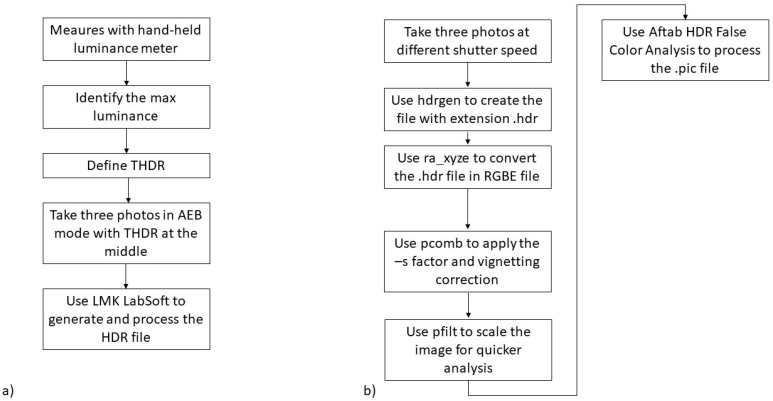
Flowchart used to create false-colour luminance map: (**a**) professional system; (**b**) low-cost system.

On the other hand, the three jpg files taken with the low-cost device are processed with the hdrgen software [11] to create the HDR file. The resulting HDR file is processed with the freely available Aftab HDR False Colour Analysis tool (Figure 4).

### 2.3. Final Setup

**Figure 5:**
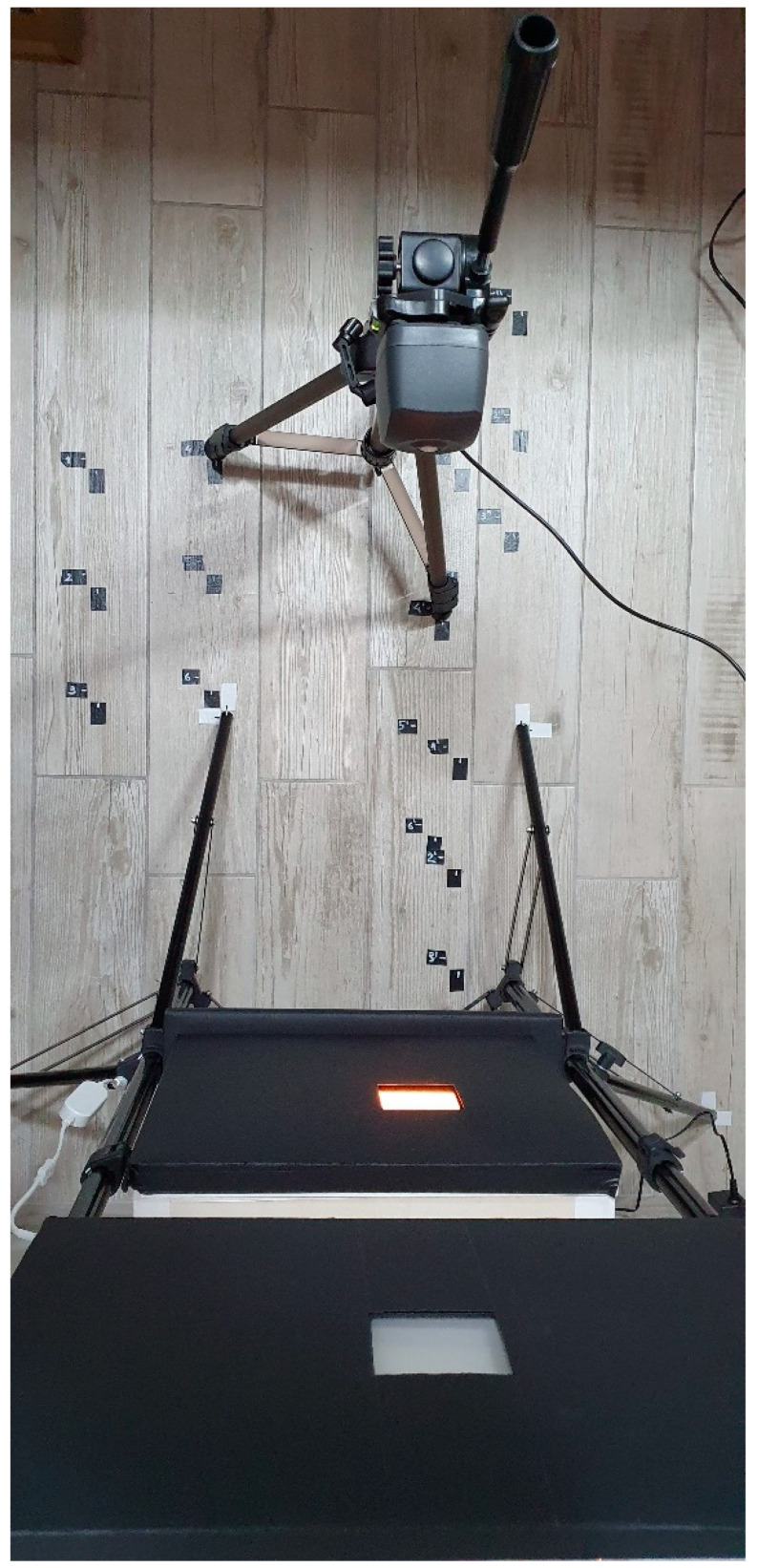
Plan view of the setup for acquiring the luminance mapping of the selected region of the LED panel and cube panel.

The final setup of the low-cost camera calibration system is shown in Figure 5.

The illuminated panels face the cameras positioned on a tripod. The tripod was also alternatively used to position the spectroradiometer (Figure 5). The vertical position is defined so that the centre of the spectroradiometer, or the centre of the segment connecting the centre of the Canon camera lens to the centre of the Raspberry cam lens, is placed at the same height as the centre of the lit panel. This configuration made it possible to collect data on luminance, which was collected in various configurations with both the professional and the low-cost camera. The same configuration also allowed the collection of data on the visual spectrum. The data are then processed to check the discrepancy in luminance mapping captured by the low-cost camera compared to the professional sensor and to see if the differences can be corrected depending on the lighting source.

**Figure 6:**
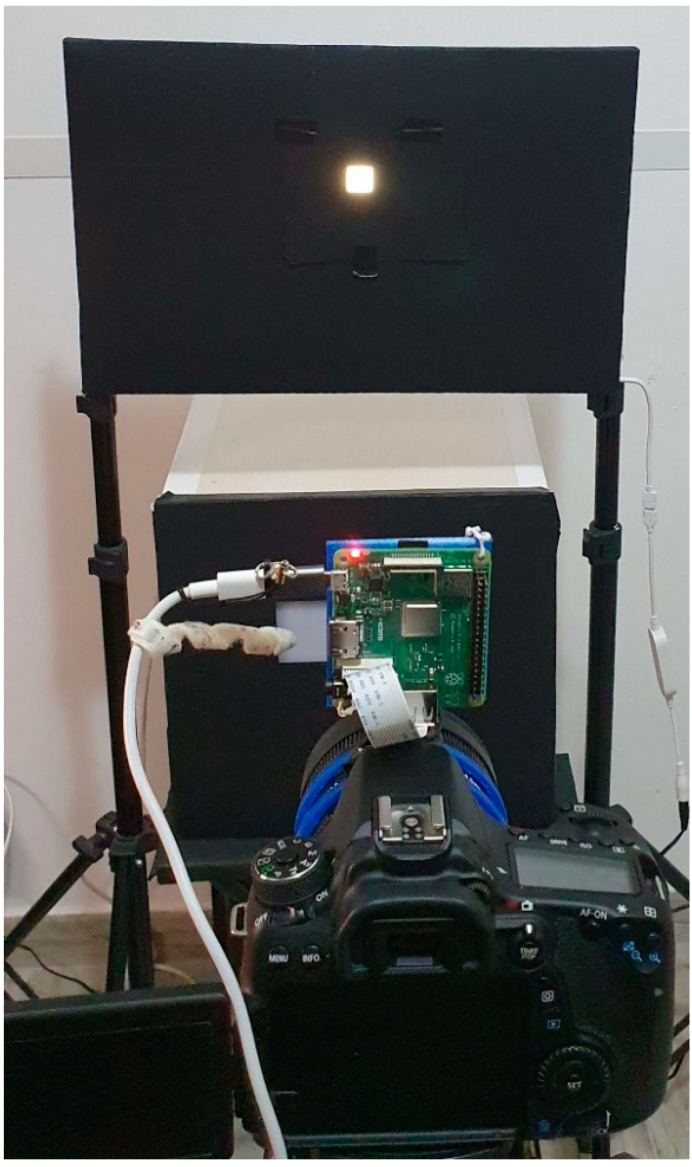
Setup for vignetting assessment: LED panel set as neutral white with 100% of intensity. Illuminated area = 2 × 2 cm^2^. Low-cost camera positioned at 60 cm from the LED panel.

Before starting the acquisition, a uniform white image was positioned in front of the camera, and a script was launched to correct via software the lens shading, also known as the vignetting effect [12,13], with a methodology often used for a microscope based on Raspberry Pi and a different type of camera with different customised lens. Then, we checked if this software correction was performed correctly. For this reason, in line with paragraph 2.3.5 of Ref [5], the setup described here was also used to verify the lens shading [13]. In this case, the tripod was positioned 60 cm from the LED panel, and the area illuminated by the LED panels was reduced to a surface of 2 × 2 cm (Figure 6).

The low-cost camera is rotated by 11.25° each time, covering the FOV of the lens, and three images at different exposure are acquired each time.

## 3. Results

### 3.1. Vignetting Assessment

**Figure 7:**
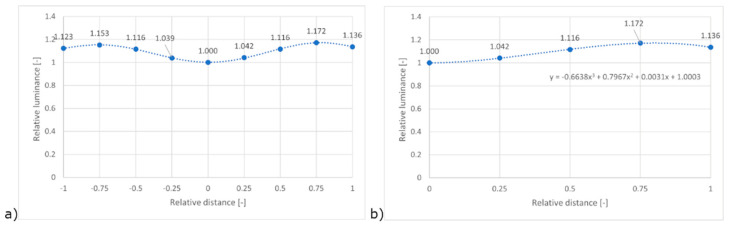
Lens shading effect pre-assessment: (**a**) relative distance 0 = centre of the image, (**b**) relative distance 1 = corner of the image.

As reported in the previous paragraph, the setup allows us to acquire three images with different exposure for the different rotation angles. By managing the derived HDR file for each rotation step with Aftab HDR False Colour Analysis tool, we determined the “luminance” value for the illuminated area. We normalised those values by considering the values in the centre of the image as equal to 1 (relative luminance, *y*-axis, Figure 7). The same approach was used for relative distance (*x*-axis in Figure 7) in line with Ref [14], where the relative distance equal to 0 refers to the centre of the image, and the relative distance of 1 refers to the corner of the image.

Figure 7 allows us to make some useful considerations:

- By applying the software correction of the low-cost camera as described above, the centre of the image records lower luminance values than those moving towards the corner of the image.
- It is possible to confirm the symmetrical distribution of the values, in line with expectations.

**Figure 8:**
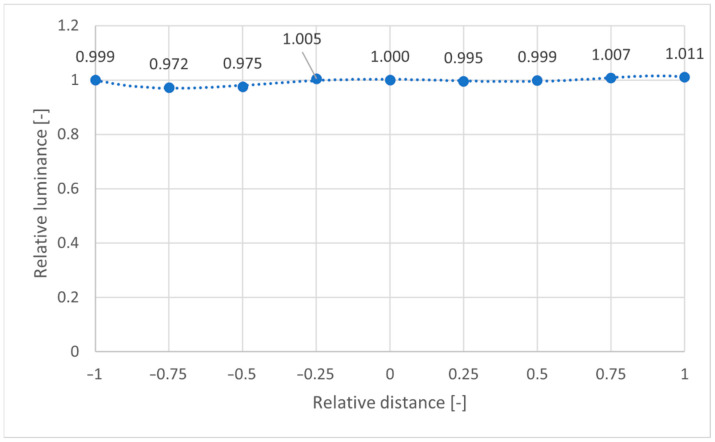
Lens shading effect post-assessment: relative distance 0 = centre of the image, relative distance 1 = corner of the image.

As confirmed by Figure 7, assuming a symmetrical distribution of the relative luminance differences, it is possible to define a calibration curve that starts from the centre of the FOV and extends to the corner. In this case, the polynomial of the third order used in the cal file of the pcomb function is composed of the coefficient reported in Figure 7b. By applying the -f function provided by pcomb, considering the cal file, it was possible to remove the spatial disuniformity of luminance, as confirmed by Figure 8.

Figure 8 shows how, effectively, the relative luminance distribution among the different relative distances almost equals 1. In the next paragraph, we focus on the difference between low-cost values of luminance and those monitored with a professional camera.

### 3.2. Panel and Cube Characterisation with Konica Minolta Luminance Reference Meter

Appendix A shows the details of the analysis of luminance resulting from applying the Konica Minolta luminance meter over the two reference sources. The data are classified, considering a string consisting of three parts (e.g., 100_C_1 ). The first is used to identify the light intensity (100% or 50%), and the second is used to identify the white type among warm (W), cool (C) or neutral (N). The third is the distance from the lighting sources: 1 = 55 cm, 2 = 30 cm, 3 = 15 cm from the lighting sources. In the case of the cube, the H, F or I letters indicate, respectively, the halogen, fluorescent or incandescent lamp used in the test without changing the intensity. Numbers 4 = 55 cm, 5 = 30 cm or 6 = 15 cm refer to the distance from the reference lighting sources. In all cases, it is possible to check the luminance distribution over the reference surfaces and identify an area of 6 × 6 cm where the monitored values are almost constant. Even though we do not know the light distribution of warm white and cool white LEDs because the manufacturer’s data are unknown (e.g., .ies file), we can test experimentally that the selected area for LED is the same for the different configurations. This is due to the geometric distribution of LED_S_ (Figure 1a), which is quite the same for warm and cool white LEDs, thus supporting the idea that there is no relevant difference in light distribution for the two types of LEDs. Table 2 summarises some details of the area luminance marked in black in Appendix A.

**Figure 9:**
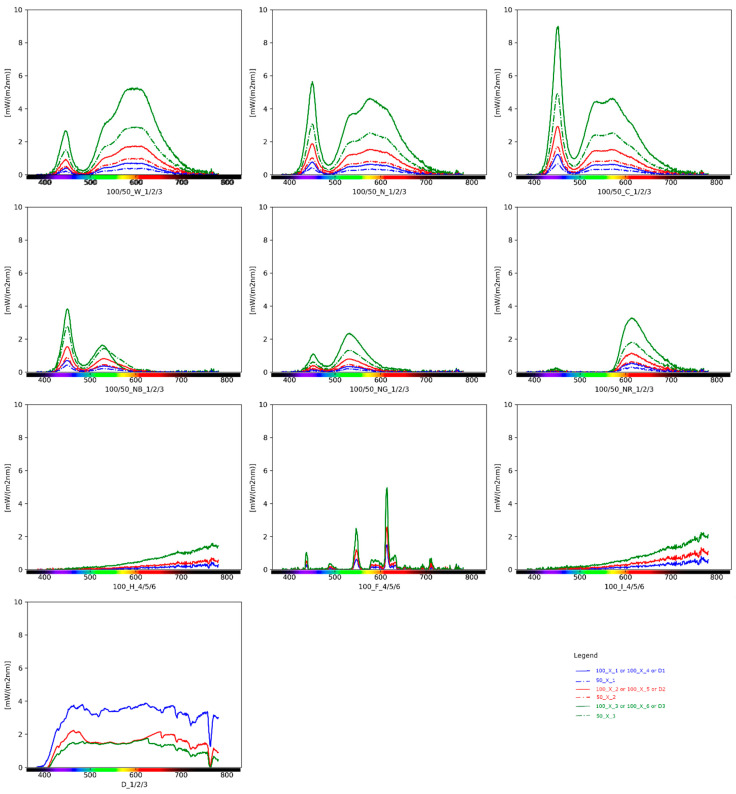
Spectrum plot differentiated for the different configurations: in the legend, X = W (Warm LED white) or N (Neutral LED white) or C (Cool LED white) or NB (Blue filter over Neutral LED white) or NG (Green filter over Neutral LED white) or NR (Red filter over Neutral LED white) or H (Halogen) or D (Daylight) or F (Fluorescent) or I (Incandescent); 100/50 = intensity; 1/2/3 or 4/5/6 = positions.

Figure 9 summarises the spectrum profiles for the different configurations considered. For better comprehension of the light source colour rendition, see Ref. [15].

### 3.3. Camera Photometer and Raspberry Camera Comparison

Table 3 reports in the second and third columns the pairwise results of the luminance evaluation with the camera photometer and the Raspberry camera. The table also reports the value of the adimensional coefficient S, the ratio between the luminance value measured with the camera phonometer and that measured with the Raspberry camera. This is a factor used in the pcomb [16] feature developed by Greg Ward to edit the starting HDR image. The fourth column reports the corrected factor of luminance by applying the S_pcomb_factor. The last three columns report data acquired with the spectroradiometer.

From Table 3, it is possible to highlight how for all the considered configurations for LED lighting, the S_pcomb factor is equal to 0.105, on average, with a minimum value of 0.08 and a maximum of 0.12. The average S_pcomb for halogen lamp configurations is 0.042, while it is equal to 0.116 for daylight, 0.136 for fluorescent and 0.045 for incandescent lamps.

**Figure 10:**
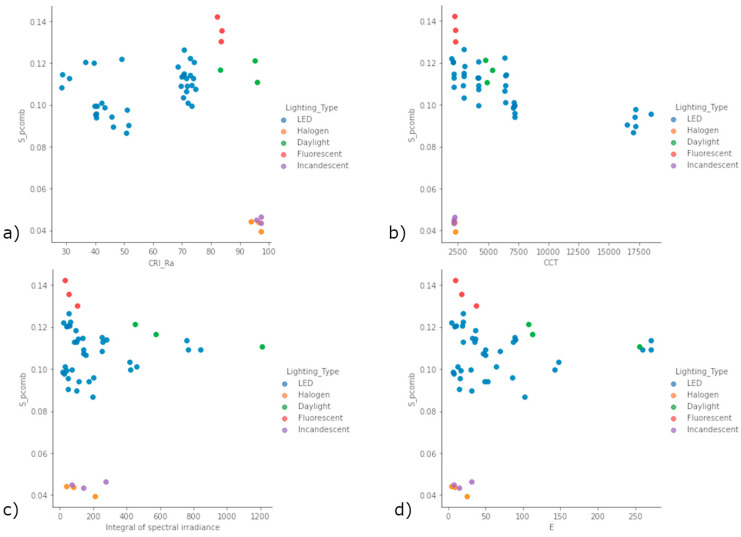
S_pcomb as a function of different parameters: (**a**) CRI_Ra; (**b**) CCT; (**c**) Integral of spectral irradiance; (**d**) E. CRI_Ra = Colour Rendering Index, CCT = Correlated Colour Temperature, E = luminance.

To answer the second question posed in the introduction, we want to verify whether it is possible to classify the S_pcomb as a function of some variables among those reported in the previous Table 3. For this purpose, Figure 10 reports S_pcomb in a two-dimensional plot as a function of different parameters characterising the different spectra.

S_pcomb does not seem to be clearly classifiable considering only one parameter among CRI_Ra (Figure 10a), CCT (Figure 10b), Integral of spectral irradiance (Figure 10c) and E (Figure 10d). It is possible to highlight how all LED configurations are characterised by a CRI_Ra of less than 81. While the Daylight and Halogen configurations are characterised by a CRI_Ra higher than 90, the difference in terms of the Integral of spectral irradiance is remarkable. For this reason, it is feasible to define a possible conditional statement that allows us to classify the lighting source in LED, Halogen, Fluorescent, Incandescent and Daylight and consequently identify the correct S factor:

- IF CRI_Ra ≤ 81 => “LED” => S = 0.105;
- ELSE IF 81 < CRI_Ra ≤ 90 & Integral of spectral irradiance< 300 => “Fluorescent” => S = 0.136;
- ELSE IF CRI_Ra > 90 & Integral of spectral irradiance< 300 => “Incandescent” of “Halogen” => S = 0.043;
- ELSE Daylight => S = 0.116.

The fairly marginal difference between halogen and incandescent lamps and the minimal difference in terms of the S factor convinced us to consider an average value for S equal to 0.043 and not to distinguish between the two types of lamps.

We can apply the proper factor S_pcomb by considering the different lighting sources.

### 3.4. False-Colour Analysis in Real Cases

Different scenarios are considered:

1. indoor space, office with daylight only (lat: 45.40182, long: 9.24962; date: 07/04/2022; time: 13:02) (CRI_Ra > 90 (96.4) and Integral of spectral irradiance > 300 (1112.5) => “Daylight” => S = 0.116);
2. indoor space, office with daylight and fluorescent lamps (lat: 45.40182, long: 9.24962; date: 07/04/2022; time: 13:22) (CRI_Ra > 90 (94.3) and Integral of spectral irradiance > 300 (1226.1) => “Daylight” => S = 0.116);
3. indoor space, industrial fabric (lat: 45.40182, long: 9.24962; date: 07/04/2022; time: 14:02) (CRI_Ra > 90 (96.5) and Integral of spectral irradiance > 300 (507.87) => “Daylight” => S = 0.116);
4. outdoor space, Ponte Coperto (PV) (lat: 45.180681, long: 9.156303; date: 06/26/2022; time: 08:50) (CRI_Ra > 90 (96.5) and Integral of spectral irradiance > 300 (4680.44) => “Daylight” => S = 0.116);
5. indoor space, living room at dusk (lat: 45.163057, long: 9.135930; date: 07/05/2022; time: 21:28) (CRI_Ra ≤ 81 (80.2) => “LED” => S = 0.105).

**Figure 11:**
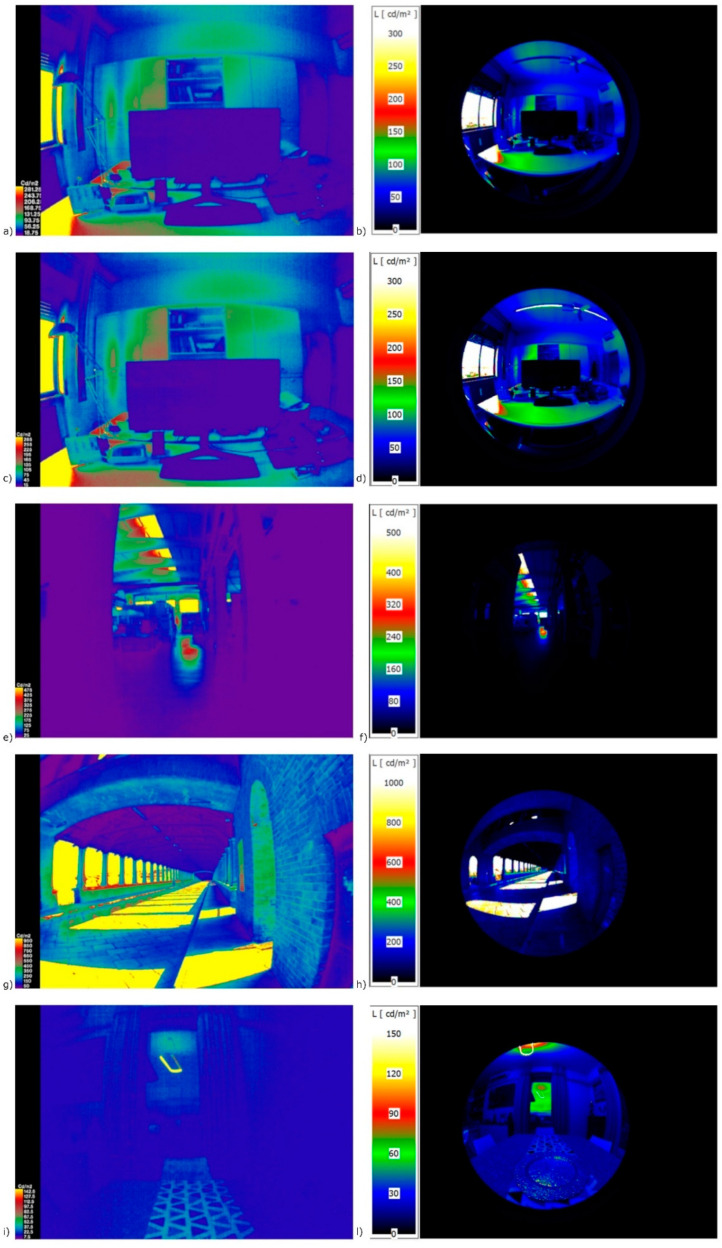
False-colour distribution of luminance in different scenarios: (**a**) office space with low-cost camera—Daylight; (**b**) office space with the professional camera—Daylight; (**c**) office space with the low-cost camera—Daylight and fluorescent light; (**d**) office space with the professional camera—Daylight and fluorescent light; (**e**) industrial fabric with the low-cost camera—Daylight; (**f**) industrial fabric with the professional camera—Daylight; (**g**) outdoor space with the low-cost camera; (**h**) outdoor space with the professional camera; (**i**) indoor space, living room at evening with the low-cost camera—LED; (**l**) indoor space, living room at evening with the professional camera—LED.

Figure 11 shows the comparison of illuminance mapping in false colour, considering the proper S factor, defined in accordance with the conditional statement used to classify the predominant light source.

It is possible to make the following considerations about the luminance distribution [cd/m^2^] with the HDRs acquired with the two systems:

- The raspicam is less resolute and also has less FoV, but we already knew this in advance;
- Even in a very low light scenario (living room at dusk), it is possible to highlight a good comparison in terms of luminance distribution, demonstrating a good criterion for selection of the light source and, consequently, the correct S factor to apply to a low-cost HDR image.

### 3.5. Glare Index Analysis

To perform glare analysis, different methods are considered, depending on the system considered.

**Figure 12:**
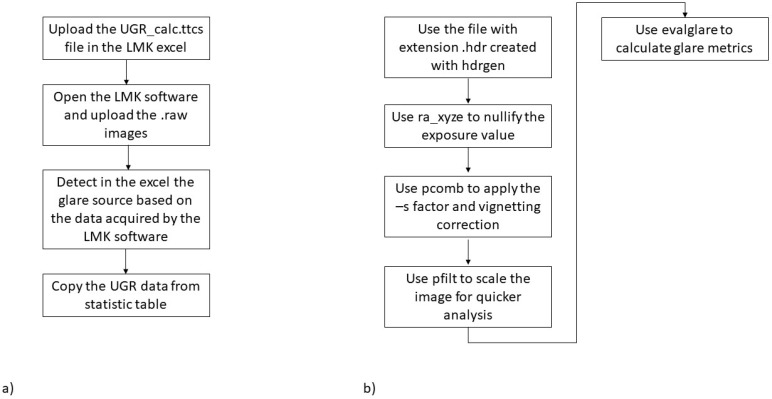
Flowchart calculates UGR: (**a**) professional system; (**b**) low-cost system.

In the case of the low-cost instrument, two different methods are used:

1. The first one considers a task area, as recommended in Ref [17]—a useful approach, especially in the case of scenarios 1, 2 and 5, where users are expected to concentrate their gaze towards a specific area. The average luminance is calculated, and each pixel exceeding this value multiplied by a default factor equal to 5 [17] is considered a glare source.
2. The second approach—especially useful in the case of walking, when users are not concentrated in a specific area—does not consider a task area, in contrast to what is reported in Ref [17]. This allows us to consider the entire area captured. In this case, a constant threshold luminance level equal to 1500 cd/m^2^ is used. This second method also considers the difference in glare assessment due to the different FoV of the acquired figures. Depending on the derived HDR image, two different approaches are considered (Figure 12).

For the HDR file generated with the professional camera photometer, the value of UGR is defined in accordance with Section 17.1.5 of Ref. [18], as synthesised in Figure 12a. Among the different methods of glare calculation reported in Ref [16], we considered the following three methods:

a. The first method—the most accurate—is based on the analysis of the overall luminance histogram and sets the first minimum after the first maximum as the luminance threshold level.
b. The second method is based on using a task area defined in the LMK LabSoft, and the average luminance of the task zone area is defined as the threshold level. The threshold level is multiplied by a factor set to 5.
c. The third method is based on manually setting a luminance threshold level—in this case, equal to 1500 cd/m^2^—for the first four scenarios, while for the fifth, a value equal to 1000 cd/m^2^ is considered.

The low-cost images are processed with ra_xyze to create the RGBE radiance file with the following code:

- ra_xyze -r -o 20220705_2128.hdr > 20220705_2128_EVinpixel.hdr
- The pcomb function is then used to apply the S factor and vignetting adjusting, as reported in the following example:
- pcomb -f vignettingfilter.cal -s 0.105 -o 20220705_2128_EVinpixel.hdr > 20220705_2128_EVinpixel_0105corr.hdr

Then, a smaller image is created with the extension pic file using the Pfilt program [19]:

- pfilt -1 -e 1 -x 1120 -y 840 20220705_2128_EVinpixel_0105corr.hdr > 20220705_2128_EVinpixel_0105corr.pic
- Pfilt -1 -e 1 -x 1120 -y 840 xxx.hdr > xxx.pic (where “xxx” expresses the name of the initial hdr file)

Then, the evalglare program [17] is used to calculate the glare metrics:

- In the case of considering the task area, the following script is used, which allows first calculating the glare indices and then saving a pic file with the highlighted task area by considering the following script:
- evalglare -T 580 350 0.7 -vth -vv 122 -vh 90 -c taskarea.pic 20220704_1302_EVinpixel_0116corr.pic
- In the case of scenarios 3 and 4, typically a walking scenario, the y position of the task area is lowered slightly and set equal to 100, imagining that the user is focused on looking at the area where they will place their feet. Then, the pic file is converted to a more useful tif file by considering the following:
- ra_tiff -z taskarea.pic taskarea.tif
- Meanwhile, in the case of considering the entire area captured, the following script is considered:
- evalglare -vth -vv 122 -vh 90 -b 1500 xxx.pic > glare_xxx.txt

The term -b allows setting the threshold luminance value in line with the third method used by the professional glare calculation method. In the case of scenario 5, this value is set to 1000 cd/m^2^.

Table 4 reports the values of UGR for the different scenarios and different methods considered above and the sensation based on the 9-point Hopkinson’s glare scale [20,21] below.

Table 4 allows some useful comments to be made. Even if we consider only the professional device, the glare evaluation in relation to the sensation scale could be very different in cases where it is not a “standard” office scenario. In particular, if we look at scenario 4 (outdoor assessment), we can see that the glare sensation calculated based on the professional device data could be “uncomfortable” or “unacceptable” or “just uncomfortable”, depending on the method used.

On the other hand, if we compare the results of methods 1 and 2 of the low-cost device for scenarios 1, 2 and 5 with the corresponding methods b and c of the professional device, we can see that there is a difference in terms of glare sensation when considering a task area (method 1 and b), while there is no difference when the whole area is evaluated (method 2 and c). Additionally, when we compare method 2 with method a for the same scenarios, there are no differences in glare sensation.

## 4. Discussion and Future Improvements

**Figure 13:**
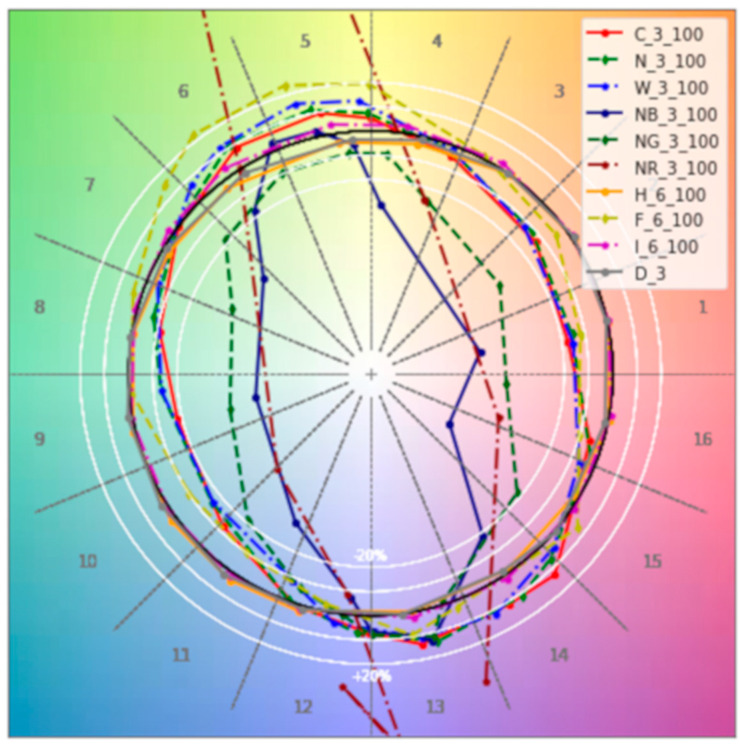
The 16 hue bins circle for different lighting sources: incandescent—I_6_100, halogen—H_6_100, daylight—D_3, fluorescent—F_6_100, warm white LED—W_3_100, cold white LED—C_3_100, neutral white LED—N_3_100, blue LED light—NB_3_100, red LED light—NR_3_100 and green LED light—NG_3_100.

The idea of performing luminance mapping with a low-cost camera is certainly not new [22,23], as the costs are more than an order of magnitude less than those of professional equipment, and the automated procedure for determining the glare index is very fast when compared to a classic manual procedure in which the photos have to be copied to the PC and then processed with dedicated software. The novelty of the proposed approach lies in the use of the DIY approach used to assess the performance of the low-cost camera, thus allowing the description and implementation of a method that is practically replicable and applicable by considering different light sources, even different from those considered in this study. With this in mind, Figure 13 shows the profiles of the light sources considered at 100% of light intensity and for the closest position to the reference light source in the 16 hue bins circle, which allow us to identify the difference in hue shift compared to a reference blackbody radiator (black line in the figure).

Some of the sources (incandescent lamp—I_6_100, halogen—H_6_100 and daylight—D_3) have a similar colour behaviour to the reference colour; others deviate by a maximum of 20% (fluorescent lamp—F_6_100, warm white LED—W_3_100, cold white LED—C_3_100, neutral white LED—N_3_100) and others still (blue LED light—NB_3_100, red LED light—NR_3_100, green LED light—NG_3_100) are intentionally very far from the black reference circle. A comprehensive overview of the colour rendering of all light sources can be found in Ref [15].

However, the approach described in this way could be repeated, and it is not surprising that more information is provided in Appendix A and in Ref [15]. This is because other researchers interested in the same aspect could replicate the easy and inexpensive instrumentation to understand how the system behaves under the action of other sources, different from those considered so far, or to consider a more in-depth study of contrasting fields, bright and dark areas side by side, which may also influence the final glare assessment due to the small size of the optical element of the Raspberry camera.

Another consideration is the presence of different light sources. In this case, the algorithm considers a total spectrum and then applies a correction coefficient that considers the predominant source. In this sense, in the case of daylight at midday, which is predominant compared to the fluorescent spectrum, the algorithm considers the total spectrum as “daylight” and assigns the corresponding S factor (S = 0.116, Figure 11b,c), while at dusk, when the daylight brightness is low and in the presence of LED light, the algorithm considers the total spectrum and assigns an S factor corresponding to the “LED” condition (S = 0.105, Figure 11h,i). The approach designed in this way allows different light sources to be considered by considering the total spectrum.

A future improvement could involve placing a surface orthogonal (or with a different angle) to the illuminated area on which different surface finishes could be applied and also investigating how the reflection effect could affect the luminance mapping of the low-cost system. This aspect is not considered in this study but does not seem to impact the overall luminance mapping and glare assessment significantly. Another improvement could be the use of a camera with higher FoV.

Another consideration regards the use of this low-cost solution for glare assessment. If we refer to the results of Section 3.4, in our opinion, it would be possible to consider a low-cost solution for indoor glare assessment in the case of office spaces (scenario 1 and 2) or home environments (scenario 5). Using a low-cost scenario for glare assessment in outdoor spaces (scenario 4) or indoor spaces (scenario 3) that differ from the classical office space requires further investigation, since, as shown, the same professional device can give different results depending on the method used.

## 5. Conclusions

A new calibration setup based on a DIY approach was proposed. The setup made it possible to perform calibration of a low-cost camera and compare the results in terms of luminance mapping with a professional DSLR camera photometer in a controlled environment but also considering real case studies.

According to the main questions formulated at the beginning of this study, we can conclude that:

- Luminance mapping can be performed using a low-cost camera if it is subjected to a time-consuming but necessary calibration process;
- The S factor of the pcomb function allows us to consider a correction factor that can be applied to the low-cost system to better match the luminance values of the professional device;
- The S factor can be differentiated by considering different light sources, and in our study, we introduce a rough algorithm that performs this;
- The calibration process could be replicated following a DIY approach to account for the different limitations/improvements, as described in the previous section.

## Figures and Tables

**Table 1:** Lighting characteristics of the reference camera photometer.

Variable	Value
Integral spectral mismatch for halogen metal discharge lamps	2–9 [%]
Integral spectral mismatch for high-pressure sodium discharge lamps	7–13 [%]
Integral spectral mismatch for fluorescent lamps	8–10 [%]
Integral spectral mismatch for LED white	5–12 [%]
Calibration uncertainty ΔL	2.5 [%]
Repeatability ΔL	0.5–2 [%]
Uniformity ΔL	±2 [%]

**Table 2:** Luminance values of the selected regions for the different configurations (3 × 3 mesh).

Configuration	Min Luminance [cd/m^2^]	Mean Luminance [cd/m^2^]	Max Luminance [cd/m^2^]
50_C	1072	1081	1089
100_C	1890	1920	1950
50_W	1046	1062	1075
100_W	1878	1918	1944
50_N	1018	1032	1048
100_N	1826	1849	1880
Cube_H	370	373	376
Cube_F	777	783	790
Cube_I	738	744	750

**Table 3:** Luminance values of the selected regions for the different configurations considering the camera data and S coefficient.

Configuration ^1^	Camera Photometer	Default Raspberry Values	S_Pcomb Factor_Mean	Raspberry Corrected Value	E	CCT	CRI _Ra	Integral of Spectral Irradiance
	[cd/m^2^]	[-]	[-]	[cd/m^2^] ^2^	[lx]	[K]	[-]	[mW/m^2^]
100_C_1	1961	17163	0.114257	1802.115	36	6471	70.4	109.36
100_N_1	1880	16645	0.112947	1747.725	35	4143	71.5	99.42
100_W_1	1944	16437	0.118270	1725.885	36	3068	68.6	97.5
100_C_2	1956	17144	0.114092	1800.12	90	6428	72.7	279.99
100_N_2	1873	16590	0.112899	1741.95	87	4215	74	257.82
100_W_2	1946	16929	0.114951	1777.545	89	3013	70.6	251.93
100_C_3	1844	16894	0.109151	1773.87	271	6430	71.8	841.3
100_N_3	1768	16168	0.109352	1697.64	260	4206	73.4	768.71
100_W_3	1836	16156	0.113642	1696.38	271	3008	69.9	758.82
100_NG_3	613	6388	0.095961	670.74	86	7195	40.4	203.81
100_NG_2	651	6535	0.099617	686.175	30	7218	40.5	72.62
100_NG_1	662	6548	0.101100	687.54	13	7120	42.2	33.13
100_NR_3	463	4270	0.108431	448.35	69	2189	28.5	253.05
100_NR_2	494	4374	0.112940	459.27	20	2170	31.1	85.18
100_NR_1	472	4264	0.110694	447.72	9	2093	39.5	41.07
100_NB_3	442	4947	0.089347	519.435	102	17022	50.8	195.23
100_NB_2	473	5279	0.089600	554.295	31	17186	46.2	100.36
100_NB_1	481	5318	0.090448	558.39	15	16515	51.4	50.36
50_C_1	1083	8851	0.122359	929.355	20	6376	72.8	62.5
50_N_1	1036	8596	0.120521	902.58	19	4174	74.1	57.67
50_W_1	1075	8506	0.126381	893.13	20	3003	70.7	56.29
50_C_2	1057	9918	0.106574	1041.39	50	6398	71.6	155.53
50_N_2	1017	9465	0.107448	993.825	47	4201	74.6	139.9
50_W_2	1058	9702	0.109050	1018.71	50	2928	69.5	140.27
50_C_3	988	9774	0.101085	1026.27	64	6408	71.9	459.01
50_N_3	937	9400	0.099681	987	142	4186	73.4	420.88
50_W_3	990	9565	0.103502	1004.325	148	3010	70.3	416.62
50_NG_3	333	3543	0.093988	372.015	49	7192	40.3	116.2
50_NG_2	353	3552	0.099381	372.96	17	7146	39.8	41.41
50_NG_1	361	3660	0.098634	384.3	7	7049	43.2	19.51
50_NR_3	253	2209	0.114531	231.945	32	2187	28.6	139.14
50_NR_2	268	2224	0.120504	233.52	11	2109	36.7	48.01
50_NR_1	270	2213	0.122006	232.365	5	1996	49.2	22.63
50_NB_3	182	1932	0.094203	202.86	53	17168	45.6	172.12
50_NB_2	252	2640	0.095455	277.2	16	18485	40.2	51.24
50_NB_1	261	2671	0.097716	280.455	8	17191	51	28.67
100_H_4	334	7566	0.044145	317.772	5	2147	93.7	40.97
100_H_5	332	7560	0.043915	317.52	9	2205	96.5	80.42
100_H_6	316	8000	0.039500	336	25	2272	97.2	212.55
100_F_4	781	5496	0.142103	747.456	10	2198	82.05	32.96
100_F_5	766	5649	0.135546	768.264	18	2268	83.8	55.51
100_F_6	757	5812	0.130282	790.432	37	2262	83.5	105.71
100_I_4	748	16657	0.044894	749.565	8	2131	95.8	71.66
100_I_5	750	17242	0.043510	775.89	15	2138	97.2	141.38
100_I_6	749	16162	0.046312	727.29	31	2221	97.3	274.74
D_1	101	912	0.110746	105.792	255	4913	95.9	1210.23
D_2	104	891	0.116723	103.356	113	5369	83.2	576.69
D_3	106	875	0.121143	101.5	107	4804	95.2	449.3

^1^ C = cool white, N = neutral white, W = warm white, NG = green filter, NR = red filter, NB = blue filter, H = halogen, F = fluorescent, I = incandescent, D = daylight. ^2^ values obtained by considering an average Scomb = 0.105 for all configurations with LED panel, 0.042 for configurations with halogen lamps, 0.116 for configurations with daylight, 0.136 for fluorescent and 0.045 for incandescent lamps.

**Table 4:** UGR values for the different scenarios and related sensation based on a 9-point scale.

	Low-Cost		Professional		
Scenario No.	Method 1	Method 2	Method a	Method b	Method c
1	20.15 (unacceptable ^2^)	20.43 (unacceptable ^2^)	21.99 ^1^ (unacceptable ^2^)	22.72 ^1^ (just uncomfortable ^2^)	21.50 ^1^ (unacceptable ^2^)
2	21.16 (unacceptable ^2^)	21.35 (unacceptable ^2^)	21.87^1^ (unacceptable ^2^)	22.43 ^1^ (just uncomfortable ^2^)	20.90 ^1^ (unacceptable ^2^)
3	16.42 (just acceptable ^2^)	24.08 (just uncomfortable ^2^)	19.37 ^1^ (unacceptable ^2^)	17.84 ^1^ (just acceptable ^2^)	16.22 ^1^ (just acceptable ^2^)
4	21.95 (unacceptable ^2^)	27.95 (uncomfortable ^2^)	25.99 ^1^ (uncomfortable ^2^)	21.56 ^1^ (unacceptable ^2^)	22.67 ^1^ (just uncomfortable ^2^)
5	0.00 (imperceptible ^2^)	2.13 ^1^ (imperceptible ^2^)	1.97 ^1^ (imperceptible ^2^)	2.08 ^1^ (imperceptible ^2^)	0.00 ^1^ (imperceptible ^2^)

^1^ weighted with solid angle Ωp [18]. ^2^ according to the 9-point Hopkinson’s glare sensation scale [20,21].

## Data Availability

Not applicable.

## Appendix A

Luminance distribution for different configurations. The selected region 6 × 6 cm is marked in black.

50_C													
y/x [cm]	0.5	3	6	9	12	15	18	21	24	27	30	33	36
0.5	440	704	655	731	678	631	568	584	636	640	613	620	479
3	691	1040	1066	1050	1030	1029	1024	1009	1011	1022	1074	1054	680
6	693	1009	1017	1033	1028	1037	1044	1052	1031	1035	1033	1019	706
9	692	983	1002	1009	1024	1058	1083	1089	1050	1027	1016	1012	665
12	654	980	1001	990	1001	1057	1072	1083	1030	1017	995	975	658
15	700	961	976	982	990	1017	1031	1036	1019	1012	1002	958	698
18	748	985	979	984	997	1018	1020	1022	1010	1016	1009	984	682
21	528	701	759	786	715	812	557	758	439	523	545	568	488
100_C													
y/x [cm]	0.5	3	6	9	12	15	18	21	24	27	30	33	36
0.5	787	1190	1231	1129	1209	1099	1071	1287	1284	1142	1161	1376	761
3	1527	1887	1882	1897	1866	1852	1846	1799	1811	1823	1918	1896	1269
6	1164	1799	1807	1858	1867	1874	1888	1870	1858	1849	1873	1792	1244
9	1126	1755	1758	1823	1855	1902	1950	1916	1853	1837	1820	1768	1141
12	1171	1766	1763	1797	1834	1887	1926	1890	1822	1805	1773	1717	1160
15	1200	1714	1725	1775	1787	1825	1848	1824	1809	1799	1767	1702	1101
18	1296	1752	1770	1766	1790	1821	1826	1802	1811	1807	1770	1748	1211
21	796	1358	1288	1343	1187	1671	1561	1288	1291	1423	1552	1278	802
50_W													
y/x [cm]	0.5	3	6	9	12	15	18	21	24	27	30	33	36
0.5	374	682	693	657	661	641	635	626	627	648	636	606	614
3	699	1028	1038	1060	1062	1023	999	989	985	999	1017	1048	654
6	643	989	1023	1043	1051	1032	1028	1019	1014	1009	1024	1020	666
9	641	974	1003	1017	1033	1056	1075	1055	1034	1021	1018	1011	627
12	654	960	986	988	1013	1043	1073	1046	1017	1008	1006	997	635
15	585	931	954	973	1001	1012	1014	1005	997	992	976	945	664
18	649	949	977	1001	1001	996	994	988	993	985	978	968	662
21	462	578	559	623	682	663	666	682	669	588	623	705	451
100_W													
y/x [cm]	0.5	3	6	9	12	15	18	21	24	27	30	33	36
0.5	719	1355	1356	1301	1287	1231	1212	1247	1314	1355	1387	1340	696
3	906	1865	1878	1908	1909	1852	1819	1796	1792	1826	1881	1916	830
6	1122	1775	1846	1878	1895	1881	1874	1848	1839	1843	1869	1847	1009
9	1037	1766	1822	1842	1883	1912	1944	1910	1871	1847	1860	1845	1092
12	1114	1771	1797	1803	1833	1899	1942	1878	1832	1812	1806	1794	1139
15	1006	1702	1754	1786	1814	1826	1821	1811	1805	1799	1779	1723	1095
18	1204	1735	1745	1797	1830	1820	1798	1800	1810	1803	1797	1808	970
21	723	1026	1062	1222	1219	1116	1085	1056	1099	1164	1028	1220	766
50_N													
y/x [cm]	0.5	3	6	9	12	15	18	21	24	27	30	33	36
0.5	497	642	705	682	665	649	697	654	664	678	718	753	544
3	573	1000	1011	1016	1012	990	978	959	964	972	1007	1021	596
6	661	952	989	1001	1011	1010	1010	996	990	989	1000	979	524
9	562	950	977	984	1008	1032	1048	1025	1007	990	986	969	534
12	576	949	960	969	992	1017	1038	1018	995	980	971	953	524
15	565	924	943	954	968	983	987	979	973	966	950	919	629
18	652	941	942	957	969	972	967	959	964	962	955	942	632
21	420	572	627	669	630	606	608	592	594	617	634	656	434
100_N													
y/x [cm]	0.5	3	6	9	12	15	18	21	24	27	30	33	36
0.5	570	996	982	1019	1003	886	910	913	1015	932	948	989	551
3	783	1786	1801	1820	1819	1780	1754	1730	1728	1750	1798	1817	1227
6	1055	1694	1769	1794	1810	1809	1810	1790	1771	1772	1794	1769	1108
9	1056	1695	1749	1770	1805	1845	1880	1841	1805	1778	1776	1742	1037
12	971	1686	1719	1737	1771	1825	1852	1826	1789	1761	1748	1710	926
15	899	1648	1690	1703	1729	1755	1765	1756	1749	1738	1708	1643	922
18	954	1673	1688	1710	1733	1737	1729	1718	1731	1726	1700	1666	964
21	656	999	1014	1042	1031	892	875	847	867	938	1005	1015	556
Cube_H													
y/x [cm]	0.5	3	6	9	12	15	18	21	24	27			
0.5	164	180	185	197	198	193	201	193	208	177			
3	251	274	282	292	295	296	295	294	288	218			
6	244	283	291	301	309	312	312	311	301	228			
9	259	297	310	320	324	329	327	324	312	228			
12	278	308	323	331	336	338	342	338	328	231			
15	304	315	328	341	351	355	355	350	342	317			
18	311	325	343	359	366	369	370	370	357	324			
21	292	329	343	358	368	375	376	375	365	290			
24	288	318	334	332	339	347	353	352	343	286			
27	247	286	303	315	320	327	331	325	311	225			
Cube_F													
y/x [cm]	0.5	3	6	9	12	15	18	21	24	27			
0.5	323	378	389	414	416	405	422	405	437	372			
3	527	575	592	613	620	622	620	617	605	458			
6	512	594	611	632	649	655	655	653	632	479			
9	544	624	651	672	680	691	687	680	655	479			
12	584	647	678	695	706	710	718	710	689	485			
15	638	662	689	716	737	746	746	735	718	666			
18	653	683	720	754	769	775	777	777	750	680			
21	613	691	720	752	773	788	790	788	767	609			
24	605	668	701	697	712	729	741	739	720	601			
27	519	601	636	662	672	687	695	683	653	473			
Cube_I													
y/x [cm]	0.5	3	6	9	12	15	18	21	24	27			
0.5	307	359	369	393	395	385	401	385	415	353			
3	501	547	563	583	589	591	589	587	575	435			
6	487	565	581	600	616	622	622	620	600	455			
9	517	593	618	638	646	656	652	646	622	455			
12	555	614	644	660	670	674	682	674	654	461			
15	606	628	654	680	700	708	708	698	682	632			
18	620	648	684	716	730	736	738	738	712	646			
21	583	656	684	714	734	748	750	748	728	579			
24	575	634	666	662	676	692	704	702	684	571			
27	493	571	604	628	638	652	660	648	620	449			
